# Inter-rater and Intra-rater Reliability of a Mobile App Method to Measure Lumbar Lordosis

**DOI:** 10.7759/cureus.55489

**Published:** 2024-03-04

**Authors:** Jency Thangasheela Gnanasigamani, Vinodhkumar Ramalingam

**Affiliations:** 1 Physiotherapy, Saveetha College of Physiotherapy, Saveetha Institute of Medical and Technical Sciences, Saveetha University, Chennai, IND

**Keywords:** low back pain, lateral view radiograph, assessment, reliability, angle, mobile app, lordosis

## Abstract

Background

Measuring the exact quantitative values of lordotic curves is a vital factor in clinical settings to prevent musculoskeletal deformities in the future. Existing lordotic assessment methods are very diverse, expensive, inaccurate, and not handy, and their availability cannot be maintained in every clinic setup.

Aim

The purpose of this research was to study the reliability of a mobile app as a feasible method to measure lumbar lordosis angle using a lateral view radiograph.

Methodology

A lateral view low back region radiograph of 58 participants was taken based on the criteria, and the experienced physiotherapists uploaded the X-ray to the mobile app and measured the lordotic angles with the support of machine learning algorithms. Descriptive statistics were used to calculate the average and dispersion of the data of the lumbar lordosis angle measured using the mobile app method (Statistical Package for the Social Sciences (IBM SPSS Statistics for Windows, IBM Corp., Version 23, Armonk, NY)).

Results

Associations between and within raters were assessed using the Karl Pearson coefficient of correlation (1.000). Inter-rater and intra-rater reliability were determined by using Cronbach’s alpha (.966) and the split-half method. The internal consistency of the mobile app was found to be good.

Conclusions

Based on our findings, we conclude that the mobile app method is reliable and useful in measuring lumbar lordosis objectively with less effort. Since the app is handy on smartphones, physiotherapists can conduct an objective lumbar lordosis assessment in clinical settings.

## Introduction

Optimal alignment of the curvature of the spine, from the neck to the pelvis, plays an essential role in distributing the load evenly during walking or in everyday life. In particular, the lumbar spine has a normal physiological curve called lumbar lordosis. This normal domed curvature distributes the weight evenly to minimize load on the vertebral body. In addition, it also increases the shock absorption function of the vertebrae and helps maintain the stability and balance of the spinal joints [1]. Furthermore, for a person to withstand the shock of abrupt forces, their spinal curvature must remain normal [2]. In addition, spinal curves are helpful to maintain erect body posture and good stability [3]. Lumbar lordosis is the ventral or inward curvature of the spine with a normal angle that ranges from 30º to 45º. If it is < 30º, it is known as hypo-lordosis, and if it is > 45º, it is known as hyper-lordosis [4]. Variation of normal lumbar lordosis angle can result in back pain and disability in the low back region [5]. Increased lumbar lordosis produces anterior tilting of the pelvis [6].

This produces changes in the lumbosacral angle that compromise the lumbar spine's stability. An increase in lordosis would cause increased stress on the posterior structures [7]. Any adjustment in the lumbar lordotic angle produces more stress on the lumbar spine and results in low back pain [8]. The prevalence of low back pain in India is found to be 6.2% [9]; 90.5% of people with low back pain have an alteration in lumbar spine alignment [10], and 70% of the population with low back pain has postural deviations in the spine [11]. Evaluation of lumbar lordosis is vital in the treatment as well as prevention of low back pain. Radiography has been recognized as the golden method to assess postural (lordosis). The use of non-invasive methods such as photogrammetry, flexicurve, spinal mice, and inclinometer is advised mainly due to radiation exposure and the cost constraints of radiography procedures. Identifying the points and their positioning on the monitor shows errors in the photogrammetry method, but it is able to offer information on the sagittal and frontal planes [12].

The greatest measurement variability is shown in flexicurve due to the inability to place the curve in the lumbar spine and difficulty in identifying anatomical landmarks [13]. Inclinometer and spinal mice have also been found to have some limitations in maintaining constant pressure during movement and difficulty in accurately palpating landmarks among participants with higher body mass indices [14]. Additionally, assessment of posture is employed by 3D methods, which provide information in transverse, sagittal, and frontal planes. However, these methods are also limited due to their high cost, unfriendly nature, and requirement for large laboratory settings [15]. Thus, posture analysis can be done with the help of dependable, simple, portable, and easy-to-use smartphone apps like "iHandy" [16]. But this app has inbuilt software and sensors, and by placing the mobile on particular anatomical landmarks, it is sensed and values are obtained. Considering the above drawbacks based on accuracy, a mobile app has been developed to measure lumbar lordosis angle with lateral radiographs based on Cobb’s method by making use of machine learning. The primary goal of the present article is to investigate the intra-rater and inter-rater reliability of a mobile app method to measure the lumbar lordosis angle.

## Materials and methods

Subjects

Lateral view low back region digital radiographs of both males and females were included. Radiographs with any pathological conditions like spondylolisthesis were excluded.

In total, 60 participants provided consent to use the digital radiographs for this present study. The sample size was determined based on the central limit theorem and references. Participants were included in this study by using the convenience sampling method. A copy of the digital radiograph was collected from the diagnostic scan center with the radiologist's consent. In that, 58 participant radiographs met the study criteria, whereas two participant radiographs were excluded due to confirmation of spondylolisthesis by the radiologist. Before engaging the participants, the Institutional Ethics Committee approved the study proposal (approval no. 001/02/2023/IEC/SMCH).

Raters

Between and within reliability of the mobile app was tested with three fixed raters. The principal investigator was the first rater (primary, rater 1) in this study, and two other raters (secondary, rater 2 and rater 3) were invited. The secondary raters were qualified physiotherapists with a minimum of four years of experience in a clinical setting. The invited raters had a one-day practice session to become familiar with the mobile app prior to the commencement of this study. The practice session included instructions on how to find landmarks on the digital radiograph of the upper border of the L1 vertebra and the upper border of the S1 vertebra. Also, they were taught how to draw the lines and how to take readings three times for a single digital radiograph. After ensuring the familiarity of the raters with the mobile app method, the assessor measured the digital radiograph of the low back region, recorded the lumbar lordosis three times, and saved the measurements in the datasheet.

Procedure

**Figure 1:**
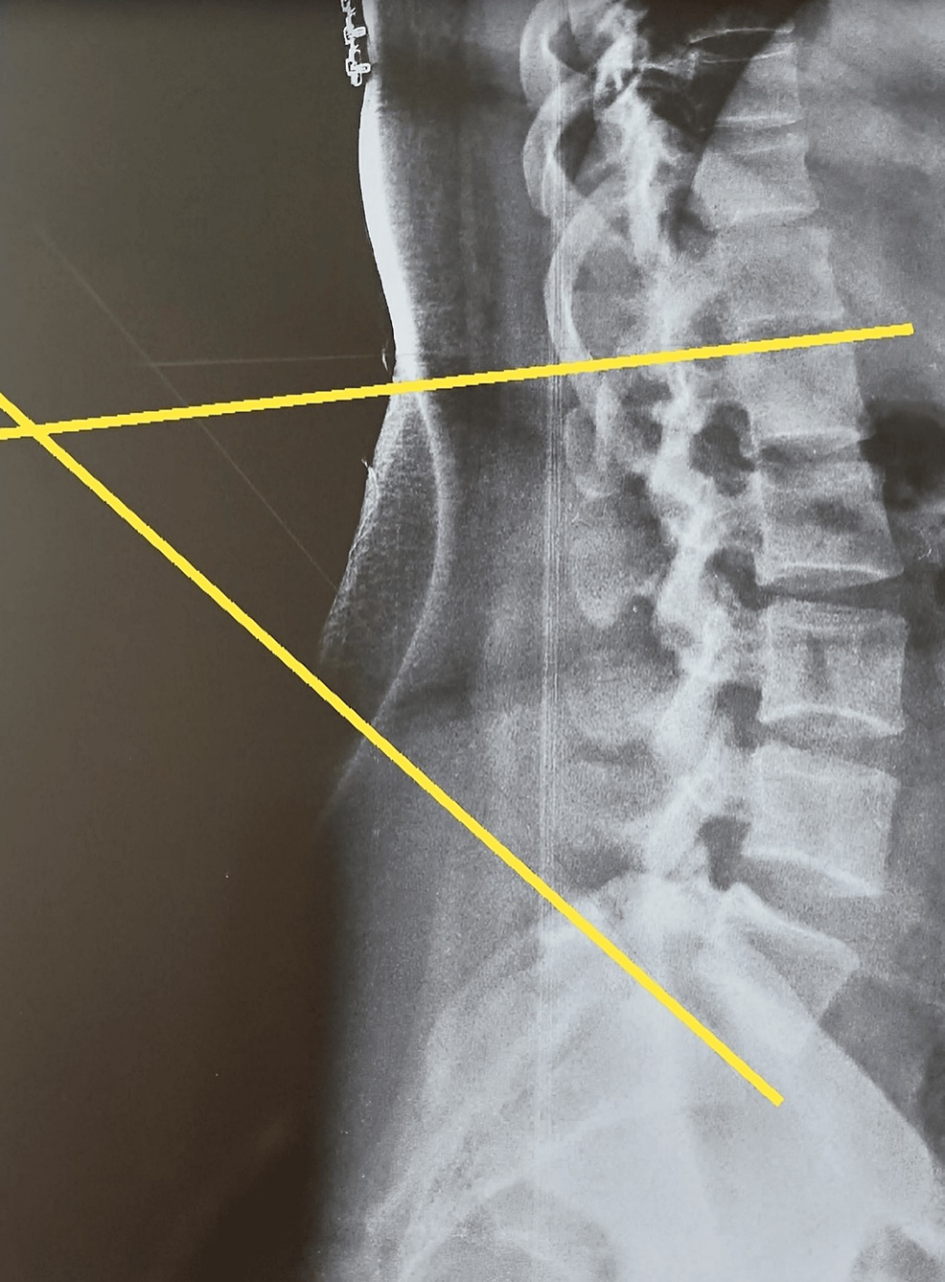
Radiograph of mesomorph participant The figure shows the drawing of the lumbar lordosis angle measurement in the mobile app.

**Figure 2:**
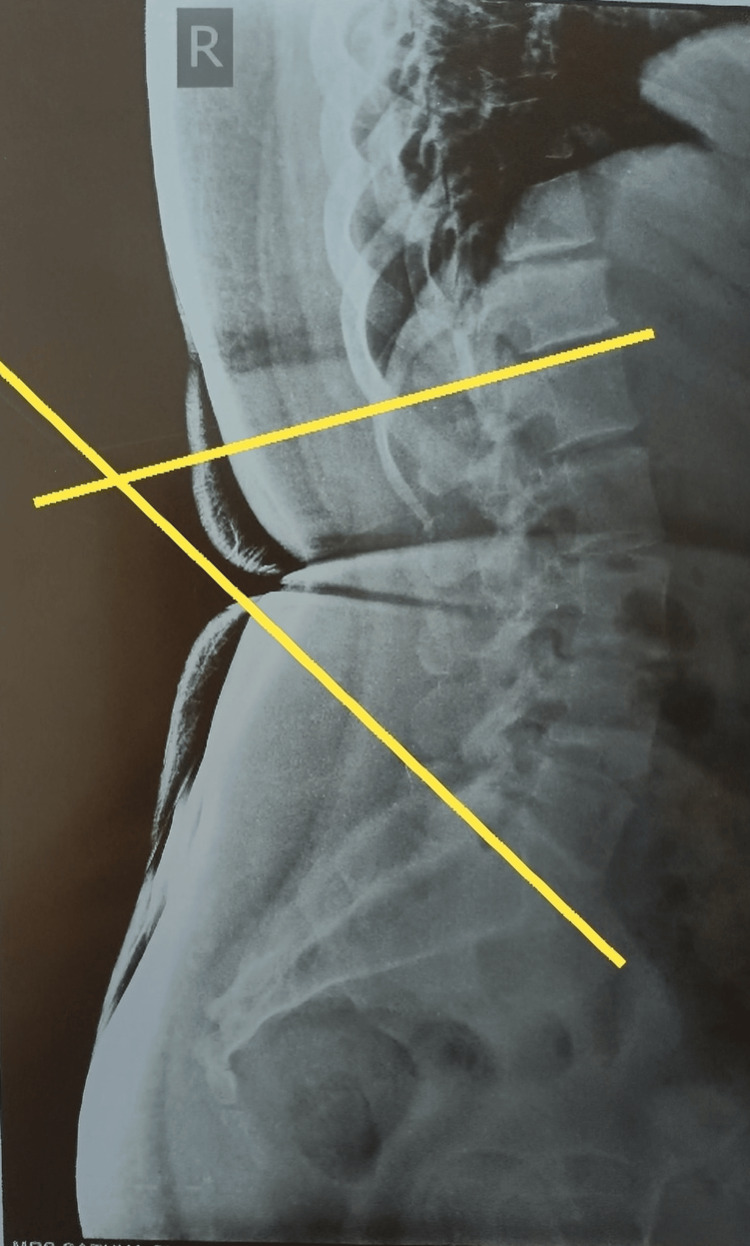
Radiograph of endomorph participant with degeneration The figure shows the drawing of lumbar lordosis angle measurement in the mobile app.

A lateral view low back region digital radiograph was uploaded onto the mobile app. The upper border of L1 and S1 vertebrae were identified. Then, a line was drawn by the rater as per the standard procedure, using their index finger to connect at one point from the upper border S1 vertebra and the upper border L1 vertebra. The measurements were taken by the rater (physiotherapist 1) three times, and then two more raters (physiotherapists 2, 3) were involved in this study. Lumbar lordosis angles were measured in the mobile app using machine learning algorithms, as shown in Figures 1-2. In both figures, the upper line denotes the marking of the upper border of the L1 vertebra while the lower line denotes the upper border of the S1 vertebra. The measured values were compared within the raters and between the raters based on clinical correlation (when it is < 30º, it is known as hypo-lordosis; when it is > 45º, it is known as hyper-lordosis).

Statistical analysis

The data was analyzed using Statistical Package for the Social Sciences (IBM SPSS Statistics for Windows, IBM Corp., Version 23, Armonk, NY). The average and dispersion of data of lumbar lordosis angle, which were measured using the mobile app method, were computed using descriptive statistics. Associations between and within the raters were assessed using the Karl Pearson coefficient of correlation. Inter-rater and intra-rater reliability were determined by using Cronbach’s alpha and the split-half method. For the 58 radiographs, in the intra-rater subset, the three measured values from the primary investigator were compared, and in the inter-rater subset, the average of the three measured values from the primary investigator (rater 1) was compared with the average of three measures from the secondary raters (rater 1 and rater 2) in order to calculate the inter-rater reliability.

## Results

Statistical analysis showed a high level of reliability in the measurement of lumbar lordosis angle using machine learning algorithms in the mobile app in between and within raters. The radiographs of the participants were of average age 42.09 (14.96) years, and the majority of the radiographs tested by the raters were female (60.3%) as compared to male (39.7%), as shown in Table 1.

**Table 1:** Participants' descriptive statistics

		Mean (SD)	Frequency	%
Age (in years)		42.09 (14.96)		
Gender	Male		23	39.7
	Female		35	60.3

**Figure 3:**
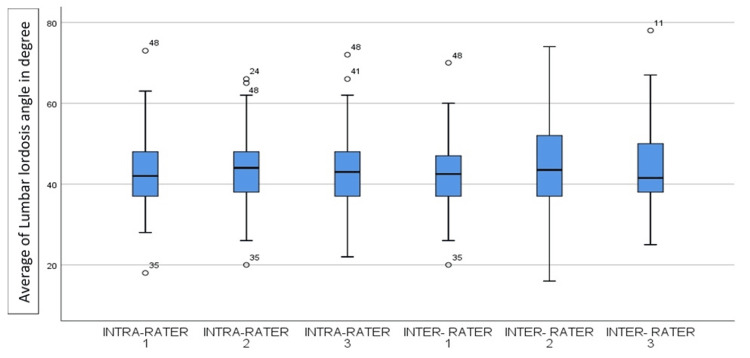
Box plot of the intra-rater and inter-rater values of raters for lumbar lordosis angle Values mentioned on the box plot are extreme values that lie beyond the average minimum and average maximum.

Figure 3, a box plot, shows the distribution of the intra-rater and inter-rater values of raters 1, 2, and 3 for lumbar lordosis angle. On observation, all the measured intra-rater and inter-rater values were evenly distributed, but the second inter-rater value showed some difference.

**Figure 4:**
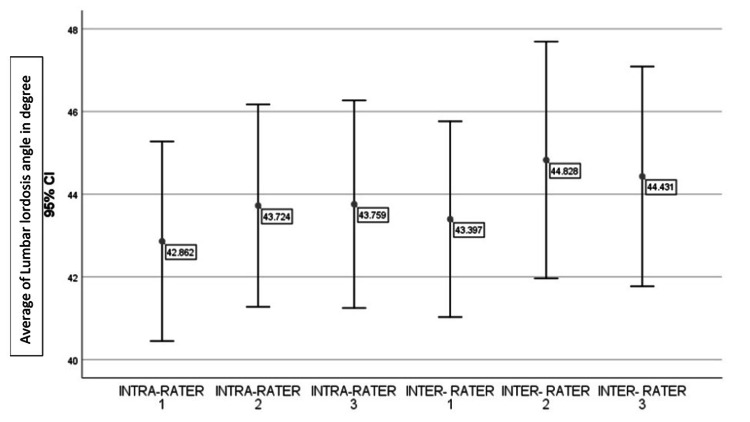
The bar graph shows the intra-rater and inter-rater reliability values of the lumbar lordosis angle

Figure 4, an error bar graph, shows the mean values of intra-rater and inter-rater reliability. The least average value is 42.862, and the maximum average value is 44.431.

The mean values are very close to each other. The line drawn from the mean values is mostly even (i.e., the collected values are evenly distributed). The graph shows 95% of the confidence interval. The relationship among the responses (measured lordosis angle) of the respondents (raters using the mobile app method) was tested using the Karl Pearson coefficient of correlation. The obtained results are presented in Table 2.

**Table 2:** Inter-item correlation matrix analysis using Karl Pearson coefficient of correlation

Raters	Intra-rater 1	Intra-rater 2	Intra-rater 3	Inter-rater 1	Inter-rater 2	Inter-rater 3
Intra-rater 1	1.000					
Intra-rater 2	.866	1.000				
Intra-rater 3	.913	.897	1.000			
Inter-rater 1	.943	.942	.976	1.000		
Inter-rater 2	.789	.801	.817	.841	1.000	
Inter-rater 3	.701	.734	.730	.773	.784	1.000

From the results, it was found that there is a significant relationship among the respondents. Reliability among the raters and within the raters was analyzed using Cronbach's alpha method. The results presented in Table 3 show that the intra-rater reliability value is (.961) and the inter-rater reliability value is (.920), both highly reliable.

**Table 3:** Intra-rater and inter-rater reliability analysis using Cronbach’s alpha Mean denotes the average of reliability, minimum denotes the least average of reliability, and maximum denotes the highest average of reliability.

	Item Means	Minimum	Maximum	N of samples	Alpha
Intra-rater	43.448	42.862	43.759	3	.961
Inter-rater	44.218	43.397	44.828	3	.920
Overall	43.833	42.862	44.828	6	.966

Based on the values scored by the same rater at different times and by different raters, it shows minimal differences in the measurement of the angle. To enrich the reliability of the tested values, the split-half method was used. In this method data were divided into two halves and the reliability was assessed. Similar to Cronbach’s alpha, the results of the split-half method also show the intra-rater reliability value of (.961) and inter-rater reliability value of (.920), as depicted in Table 4, which are considered highly reliable and strongly recommended.

**Table 4:** Intra-rater and inter-rater reliability analysis using the split-half method Mean denotes the average of reliability, minimum denotes the least average of reliability, and maximum denotes the highest average of reliability. ^a^: The reliability data collected from intra-rater 1, 2, and 3; ^b^: The reliability data collected from inter-rater 1, 2, and 3; Part 1: Observations from intra-rater 1, 2, and 3; Part 2: Observations from inter-rater 1, 2, and 3

		Mean	Minimum	Maximum	N of samples	Alpha
Item Means	Part 1	43.448	42.862	43.759	3^a^	.961
	Part 2	44.218	43.397	44.828	3^b^	.920
	Both Parts	43.833	42.862	44.828	6	

## Discussion

The primary focus of this study was to find out the intra-rater and inter-rater reliability of the mobile app method to measure lumbar lordosis with lateral radiographs. The lumbar lordosis or spine curvature has been measured using various methods in previous research: photogrammetry, flexicurve, spinal mice, inclinometer, the iHandy smartphone application, and 3D radiographic imaging. In this present study, the relationship among the measured lordotic angles with raters (using the mobile app method) was tested using the Karl Pearson coefficient of correlation. It showed a perfect positive correlation among the raters (1.000) and within the raters (1.000). Based on the obtained results analyzed by Cronbach's alpha (.966) and split-half method (.961), the reliability of the mobile app method is found to be good.

Likewise, a previous study showed high concordance with intraclass correlation coefficient (ICC) 0.83 and 0.78, which were reported between flexicurve and radiographic measurement (r = 0.60) in between and within evaluators by de Oliveira et al. [17]. However, the study had a few limitations associated with flexicurve measurements, including the following: inability to place the flexicurve in the lumbar region, difficulty in identifying the vertebrae markings in the lumbar area, and angle error measurements due to insufficient practice of angle calculation methods [13,17]. Whereas the present study found easy and clear anatomical landmarks of the spine since digital X-rays were used with the support of a mobile app that measures the lumbar lordotic angle, and they are highly reliable.

On the other hand, measuring lumbar lordosis with flexicurve and comparing it with the X-ray method demonstrated strong intra-tester (ICC = 0.89) and inter-tester (ICC = 0.82) dependability. Construct validity of the flexible ruler method was reported by Seidi et al. [18]. In contrast, the flexible ruler shows poor reliability (ICC = 0.31) between the raters, which was addressed by Filho et al. [19]. Another study by Russell et al. revealed that the lumbar lordosis angle measured by the spinal mouse unit made of wood in 50 subjects wearing shoes with high heels and not wearing shoes with high heels shows concordance with ICC > 0.99. However, spinal mice validity for the angle of lumbar lordosis measurement in the sagittal plane has not been done yet [20]. Individual measurement variations in the angle of lordosis by spinal mice were informed by authors from their findings [21,22]. Because the spinal mouse measurements are taken with a skin surface device, they follow the distribution of subcutaneous tissue along the line of posterior elements, primarily in the lumbar region [23,24]. Application of the spinal mouse was limited by being conducted only among healthy or asymptomatic participants. Further, the accuracy of measurements might be challenging to carry out in symptomatic patients over the skin surface [25,26].

Measuring Cobb’s angle in photogrammetry technique and its validity compared to the radiography method was evaluated by Drzał-Grabiec et al., who showed a strong correlation between the photogrammetry method (r = 0.88) and Cobb’s angle (r = 0.30) [27]. However, the main constraint related to this technique is the prospect of errors while labeling and tagging landmark positions on the monitor [12,27]. Since using digital X-rays maintains clarity and lines drawn from the anatomical landmarks are accurate, measured angles would be accurate and less time-consuming.

When utilizing the iHand level application on an iPhone model 4, the ICC values for between and within concordance were 0.96 and 0.81, respectively (iPhone is a trademark of Apple Inc., Cupertino, USA). It has an angle-indicating digital display and an accelerometer [28]. Comparable concurrent validity (r = 0.86) was found between the iHandy and the bubble inclinometer. Similar to this, Koumantaki et al. found that intra-rater reliability for males and females was 0.96, and 0.93, respectively [14].

Another study used 3D radiographic imaging to assess spine curvature in asymptomatic subjects; the between and within concordance values were 0.97-1.00 and 0.95-1.00, respectively [29]. The consistency increased and processing quickened as the user became acquainted. The study reported the benefits of the 3D imaging method in gaining a greater insight into the 3D perspective of stance [30]. Similarly, in the present study, the intra-rater reliability value was (.961), and the inter-rater reliability value was (.920), indicating that the mobile app method is highly reliable. The reliability values of the above-quoted studies are lower than in this present study using the mobile app. In clinical settings, a mobile application offers a rapid, simple-to-use, precise, and alternative approach to evaluate sagittal plane spine curvature. Since the angle is measured through X-rays, expenses are less, availability and accuracy can be maintained, and it is easier for reassessment to have a clarification in the assessment process. In summary, the test-retest and intra-rater reliability of the results presented in this study shows that the mobile app method can be used in the initial evaluation of spinal curvature in the sagittal plane for diagnostic purposes in clinical setups. However, the study may have limitations because the number of subjects in the inter-rater and intra-rater subsets was quite small. Although this study demonstrates high reliability for the mobile app method, it does not demonstrate its validity; hence, further research is needed.

## Conclusions

Based on the findings, the study concludes that the mobile app method is useful in measuring lumbar lordosis in less time, with more accuracy, and is handy. Since this application is available on smartphones, with the support of machine learning, it will be quite useful in clinical settings for physiotherapists and other healthcare professionals to have an objective assessment tool for assessing lumbar lordosis.
